# Safety assessment of rice bran oil in a chicken embryo model

**Published:** 2016

**Authors:** Atefeh Araghi, Saeed Seifi, Reza Sayrafi, Parisa Sadighara

**Affiliations:** 1 *Faculty of Veterinary Medicine, Amol University of Special Modern Technologies, Amol, Iran*; 2 *Department of Environmental Health Engineer, Food Safety Division, Faculty of Public Health, Tehran University of Medical Sciences, Tehran, Iran*

**Keywords:** *Safety assessment*, *Rice bran oil*, *Malformations*, *Oxidative stress*

## Abstract

**Objective:**

Rice Bran Oil (RBO) is extracted from the outer layer of rice. Little information is available regarding its safety. The present study was conducted to assess its safety in chicken embryo model.

**Materials and Methods:**

RBO was injected on day 4 of incubation of chickens. The tissues and serum samples were collected. Oxidative stress parameters in the liver, kidney and brain and biochemical parameters of serum were measured. The deformities were also investigated.

**Results:**

The changes in the liver enzymes activity were not statistically significant. There was significant decrease and increase in lipid peroxidation and glutathione level, respectively. It is suggested that RBO is a natural antioxidant source. Low-density lipoprotein cholesterol (LDL) also decreased. No abnormal findings were observed in the chickens.

**Conclusion:**

No toxic effect was observed following RBO administration in chicken embryos. This study showed that RBO is not a safety concern.

## Introduction

Rice is the seed of *Oryza sativa* or *Oryza glaberrima*. It is the main food in many countries in Asia. Rice bran oil (RBO) a byproduct of rice processing industry is extracted by innovative technologies and has mild flavor, high smoke point, good stability and no adverse effect that make it a suitable alternative for other widely-used oils for industrial and culinary purposes (Liang and Ying 2014). The high smoke point prevents fatty acid breakdown at high temperatures. RBO decreases cholesterol (TC), low-density lipoprotein cholesterol (LDL-C), and triglyceride (TG) concentrations (Berger et al., 2004; Kim, 2005). It is efficient in preventing heart attack. Many studies have shown that bioactive compounds in RBO have therapeutic effects against diabetes mellitus (Posuwan, 2013), cancers (Shih, 2011), brain aging and neurodegenerative diseases (Stephanie, 2013) by reducing oxidative stress.

There is an increasing interest in consuming health-related products, and RBO has many beneficial properties as rice is the major crop in many countries thus RBO has the potential to become a popular edible oil. The hypocholesterolemic effect of RBO was approved in human. Its hypocholesterolemic effect is equal to corn, safflower and sunflower oils. This effect will be greater if given for a longer period (Sugano and Tsuji, 1997). 

RBO is unconventional oil, but there is a clear increase in consumption of this product. Therefore, it is very important to evaluate any possible toxicological risk of this product. The avian embryos particularly fertile chicken eggs posses characteristics which make them suitable for toxicological studies (Pourmirza, 2008). The aim of the present study was to investigate the toxic effects of RBO on chick embryo.

## Materials and Methods

**Animals and chemical**

Thirty eggs were divided into three groups. The eggs were candled on the day 4 and injected with edible grade RBO. RBO was from Kasisuri Co. Ltd (Thailand).

Next, 0.1 ml of RBO was injected *into* the *chorioallantoic* membrane (group a) and the same volume were also injected into the egg yolk (group b). Chorioallantoic has a number of useful properties, particularly with respect to toxicity testing (Seabra and Bhogal, 2010). The injection sites were closed with paraffin and the eggs were incubated at 37.5±0.1°C and 50–60% relative humidity. The eggs were candled the day after injection and thereafter every 48 h for checking dead embryos. The experiment was terminated on the day 20 of incubation. The embryos were checked for external malformations. Blood samples were collected. The brain, kidney and liver were removed and washed with isotonic saline. Serum and tissues were stored at -20°C until used for toxicity assessment.

**Measurement of oxidative stress parameters**

*Measurement of lipid peroxidation*

The formation of thiobarbituric acid in organ samples was assessed for evaluation of lipid peroxidation according to an original method (Sicinska et al, 2006). Briefly, the supernatant of the tissue homogenates was mixed with 20% trichloroacetic acid and the mixture was centrifuged. Then, thiobarbituric acid was added to the supernatant and heated. The absorbance of the supernatant was measured at 532 nm. The values were expressed as nmoles malodialdehyde, using a molar extinction coefficient of 1.56 × 10^5^ M^-1^ cm^-1^.

*Measurement of total GSH*

The glutathione contents were measure according to a previous study (Gibson et al. 1998). The tissues were rinsed three times with PBS. Tissue solutions were mixed with 20% trichloroacetic acid. Samples were centrifuged. The supernatants were mixed with 4 vol of Tris-buffered saline (TBS). Then, 1mM DTNB was added to the samples and incubated for 30 minutes. The absorbance was read at 412 nm.

*The ferric reducing antioxidant power* *assay*

 Total antioxidant capacity was determined by the ferric reducing antioxidant power (FRAP) method. First, the stock solutions including 300 Mm acetate buffer, 10 mM TPTZ (2,4.6-tripyridyl-s-triazine) solution in 40 mM HCl, and 20 mM FeCl3.6H2O solution were prepared. 

The fresh working solution (FRAP reagent) was prepared by mixing 25 ml acetate buffer, 2.5 ml TPTZ solution, and 2.5 ml FeCl3.6H2O solution. The samples and deionized water were mixed with 3 ml of the FRAP reagent and allowed to react for 5 min in the dark. The changes in absorbance at 593 nm are related to the total reducing power of antioxidants of tissues (Morales and Paredes, 2014).

**Biochemical parameters**

*Measurement of liver enzymes*

Alanine aminotransferase (ALT), aspartate aminotransferase (AST), and Alkaline phosphatase (ALP) activities in serum using by kits purchased from Teb Gostaran Hayan (Tehran, Iran) using UV absorbance.

*Lipid analysis*

Low density lipoprotein (LDL) and total cholesterol (TC) were measured in serum**.**

*Survey of morphological changes of embryo*

The use of chicken embryos has been approved for studying malformations caused by xenobiotics. Embryos were observed for morphological changes. The gross lesions and skeletal fetal alterations, edema and the rate of growth were surveyed. The development of control embryos was compared to (a) and (b) embryos. The weight of embryos and growth retardation were carefully recorded. The failure of retraction of yolk sac was also surveyed. 

**Statistical analysis**

Statistical differences between groups were analyzed using Student’s t-test by SPSS software. The difference is less than p≤ 0.05 was considered significant. Data values were expressed as mean±SD.

## Results

**Measurement of oxidative stress parameters**

Level of lipid oxidation in samples following RBO treatment was shown in Table 1. The level of lipid oxidation was significantly different in kidney, brain and liver. The level of lipid peroxides decreased in treated samples.

In present study, the major antioxidant defense agent, glutathione significantly increased in group (b) as compared to control (p=0.03) in the liver. RBO also significantly increased glutathione level in the kidney. Statistical analysis showed that the p value between control and (a) group was 0.02 and (b) group was 0.007.

The levels of ferric reducing capacity in control group were significantly different from those of group (b) (p<0.05) in the brain, kidney and liver.

**Table 1:** Levels of oxidative stress parameters in the liver, kidney and brain

		**Level of lipid peroxidation** **(nmol/** ** g tissue** ** )**	**GSH** **µmol/ g tissue**	**ferric reducing capacity** **mmol/g tissue**
**Control **	Liver	0.29±0.07	0.07 ±0.02	1.6 ± 0.3
**Group a**		0.19±0.07*	0.11 ±0.03*	2.01± 0.3*
**Group b**		0.18± 0.05*	0.12 ±0.03*	2.793± 0.4*
**Control **	Kidney	0.58±0.19	0.09 ±0.04	1.8 ± 0.54
**Group a**		0.33±0.17*	0.34 ±0.21*	2.6 ± 0.52*
** Group b**		0.30±0.2*	0.44 ±0.21*	2.7 ± 0.5*
**Control **	Brain	0.52±0.2	0.06±0.02	0.71 ±0.14
**Group a**		0.21±0.09*	0.06±0.02	0.81 ±0.15
**Group b**	0.15±0.04*	0.12±0.03*	1. 27 ±0.1*

Glutathione** (**GSH**)**,

* p<0.05

**Biochemical parameters**

The general signs of hepatoxicity are alterations in enzyme activity of liver. The enzyme activity was not changed.

 The effect of RBO on blood levels of TC and LDL was surveyed. The level of LDL decreased 19 % in group a compared to control group (Table 2).

**Survey of morphological changes of embryo**

Macroscopic changes were also surveyed. Macroscopic lesion and developmental abnormalities were not observed in the treated groups. The results are presented in Table 3. The survival rates in the treated groups were high and similar to control. The rate of failure of retraction of yolk sac was 10% in control group, 20% in group a, and 10% in group b.

**Table 2:** Biochemical parameters in serum

	**The mean of enzyme activity (U/L)**			LDL	TC
	ALT	AST	ALP		
**Control**	5.200± 6.301	175.20±10.756	2049.0±503.29	156.20±80.176	352.60±90.007
**Group a**	6.600±2.302	212.40±43.247	1925.6±309.89	127.00±103.72	329.00± 98.549
**Group b**	4.000± 2.236	231.00±34.525	2365.6±1272.9	144.20±70.578	352.40±22.601

Alanine aminotransferase (ALT), aspartate aminotransferase (AST), Alkaline phosphatase (ALP), Low density lipoprotein (LDL), and total cholesterol (TC). There was not significantly different between groups.

**Table 3:** Embryotoxic effects of RBO

	**Survival rates %**		**embryonic body weight** **(gr)**	**Malformations **						
				**Incomplete skull**	**Beak deformities**	**Abnormal eye size**	**Neck /head edema**	**Clubbed feet**	**Hemorrhage feathers**	**Failure of Retraction of Yolk Sac**
**Control**		90%	46.24± 3.9	-	-	-	-	-	-	10%
**Group a**		80%	44.2± 3.7	-	-	-	-	-	-	20%
**Group b**		90%	46.87± 2.6	-	-	-	-	-	-	10%

## Discussion

In this study, we carried out toxicological study of RBO with regards to its probable organ (liver, brain and kidney) damages in a chicken embryo model. Embryos have several advantages as compared to adult animals as research models. These models represent valuable assets to both fundamental and applied research and are used in various research areas including developmental biology, physiology and toxicology (Seabra and Bhogal, 2010). 

Oxidative stress results from excessive levels of reactive oxygen species (ROS) and damages macromolecules such as DNA, lipids, proteins and carbohydrates. ROS attacks polyunsaturated fatty acids and causes lipid peroxidation. Malondialdehyde (MDA) is a product of lipid peroxidation that has been used as an indicator of oxidative damages. This component is highly reactive and can damage biological molecules such as DNA. MDA is mutagenic and tumorigenic (Zhang et al. 2001). The brain is extremely susceptible to lipid peroxidation because of the accumulation of polyunsaturated fatty acids. Polyunsaturated fatty acids are vulnerable to free radical damages. The level of lipid peroxidation markedly decreased in the other organ (Table 1). It has been suggested that these results could be related to antioxidant properties. Therefore, RBO is a resource of protection brain against oxidative stress. 

The oxidative stability of organs depends on the level of antioxidants. The presence of antioxidants has an important role in the prevention of organ toxicity. These results demonstrated that RBO has antioxidant activity which is in agreement with other studies. RBO possesses antioxidants such as tocopherols, gamma-oryzanol, and phenolic compounds (Chotimarkorn, et al. 2008). The depletion of glutathione (GSH) by formation of GSH conjugates was associated with increased toxicity (Lee et al. 2010). GSH content is considered as a critical factor in protection against embryotoxicans (Surai, 1999). During embryonic development, glutathione levels continuously decrease. Oxygen is required for embryo development. However, the use of oxygen also poses a potential hazard via formation of reactive oxygen species (ROS). ROS at high concentration damage macromolecules and alter their biological functions. Glutathione is involved in the protection against ROS damages. It detoxifies ROS (Ufer and Wang, 2011). ROS can be also removed by other antioxidants. Therefore, glutathione will be preserved. In this study, glutathione level in the kidney, liver, and brain were increased compared to control group. This result concludes that, the antioxidant agents in RBO enhance the level of glutathione via ROS detoxification. These results also confirmed the antioxidant capacity of RBO. An imbalance between free radical generation and antioxidant capacity in the embryo could result in permanent damages during sensitive period of their development. This time requires a considerable degree of antioxidant against peroxidation (Surai, 1999). 

Enzymes like ALT, AST and ALP are the main indices of liver injury (Liu et al. 2012). Serum ALT activity is the laboratory indicator of hepatotoxicity. Damaged hepatocytes release ALT and AST into serum. ALP activity is additional conventional biomarker of liver function (Ozer et al, 2008). Therefore, in this study these general marker enzymes of liver were measured. Increases in plasma levels of these enzymes are considered as a sign of hepatotoxicity. The enzymes activities are not significantly changed compared to control group. The liver plays an important role in metabolism of food and is susceptible to food-induced toxicity. It is also possible that this product does not induce hepatotoxicity. 

In this study, serum lipids were evaluated. There were differences in LDL levels between groups. Results similar to our data have also been reported. Studies on experimental rats demonstrated RBO hypolipidemic effect. The hypocholesterolemic effect of RBO was also approved in human. Its hypocholesterolemic effect is equal to corn, safflower and sunflower oils. (Sugano and Tsuji, 1997). 

External malformations in chick embryos of RBO are shown in Table 3. This model provides information on embryonic survival rates and growth retardation. Hemorrhages could be due to vascular damage. Chickens did not exhibit hemorrhages, hematomas, and morphological changes. Chick embryo test for detection of teratogenic effects is reliable. This test evaluates morphological disturbances during development (Pourmirza, 2008). The results of this study suggest that RBO does not induce external structure abnormalities in the developing chick. The survival rate of embryos is similar to control embryos. Abnormalities such as weight loss were not observed.

The safety assessments and toxicological studies should be conducted for any kind of food. This evaluation provided basic information regarding the safety of RBO. The results of this study demonstrated that RBO showed no especial toxicity in chicken embryo model. This oil might be safe for human consumption. Other comprehensive toxicological evaluation should be conducted on this unconventional oil.

## Conflict of interest

The authors declare no conflicts of interest.
